# A Multifaceted Analysis of Self-Medication With Antibiotics in South-East Delhi: A Mixed-Method Study Among Adults Who Self-Reported Antibiotic Use in the Past Three Months

**DOI:** 10.7759/cureus.84623

**Published:** 2025-05-22

**Authors:** Roshan Jabeen, Aqsa Shaikh, Richa Gautam, Nusrat Nabi, Eram Sultana, Aysha Bano

**Affiliations:** 1 Community Medicine, Hamdard Institute of Medical Sciences and Research, New Delhi, IND; 2 Pharmacology, Hamdard Institute of Medical Sciences and Research, New Delhi, IND

**Keywords:** amr, antimicrobial resistance, knowledge-attitude-practice, mixed-method study, prevalence, qualitative study, self-medication with antibiotics, sma

## Abstract

Objectives

The misuse of antibiotics fuels antimicrobial resistance (AMR), rendering treatments ineffective, increasing healthcare costs, and increasing mortality. This study estimated the prevalence, patterns, and drivers of self-medication with antibiotics (SMA) among adults, alongside exploring the reasons behind this practice.

Methodology

This community-based mixed-method study ran from January 2023 to June 2024. Simple random sampling selected 250 participants for the quantitative component, and purposive sampling chose 19 participants for the qualitative component. Descriptive statistics, cross-tabulation, and logistic regression were executed using SPSS (IBM Corp. Released 2019. IBM SPSS Statistics for Windows, Version 26.0. Armonk, NY: IBM Corp). Thematic analysis of focus group discussions (FGDs) was done using NVivo 14 (Lumivero 2023, *NVivo *Version 14, Denver).

Results

The prevalence of SMA was 36.4%(95% confidence interval (CI): 31.4-41.4), with 63 (69.2%) participants self-medicating at least once in the past three months, primarily for cold and flu-like symptoms (29, 31.86%) and fever (27, 29.69%). Key predictors of SMA were marital status, religion, education level, socioeconomic class, presence of chronic disease, knowledge, and attitude. Four (4.4%) participants experienced adverse drug reactions, 167 (66.8%) had inadequate knowledge about antibiotics, with only 13 (5.2%) aware of AMR, 142 (56.8%) had a favorable attitude towards SMA, and 171 (68.4%) followed appropriate practices. FGDs identified convenience, trust in local chemists, economic constraints, misinformation, long waiting times, limited healthcare access, and community acceptance as SMA drivers.

Conclusion

The study found that the prevalence of SMA in South-East Delhi surpasses rates in other regions of the country and signals a critical public health concern. To address SMA and mitigate AMR, authorities should enforce stricter regulations on over-the-counter antibiotic sales and implement targeted community education programs to enhance awareness of rational antibiotic use and AMR risks. These interventions, by improving regulatory oversight and knowledge, can effectively reduce SMA and safeguard antibiotic efficacy.

## Introduction

Self-medication (SM), as defined by the World Health Organization (WHO), involves treating self-recognized conditions without prior consultation with a health professional or reusing previous prescriptions [1].

Antibiotics are among the most commonly self-medicated drugs, with the WHO reporting that over 50% of antibiotics are bought and used without a prescription [2-4]. The global prevalence of self-medication with antibiotics (SMA) ranges from 7.3% to 85.59%, with an overall incidence of 42.64% [5]. Between 2000 and 2015, global antibiotic consumption, measured in defined daily doses (DDD), increased by 65%, largely driven by low- and middle-income countries like India, China, and Pakistan [6]. In India, antibiotic consumption doubled from 3.2 to 6.5 billion DDDs during this period despite a low per-capita rate compared to other countries. India relies heavily on broad-spectrum antibiotics, which the WHO advises for restricted use [4]. A study conducted by Juneja et al. in 2020 in Maharashtra reported that the prevalence of SMA is 16.1%, and the major reasons were convenience and lack of time, past experiences with similar illnesses, ignorance of disease severity, confidence in avoiding doctor visits, lower costs, and recommendations from others were the commonly cited reasons for self-medication [7]. 

Antimicrobial resistance (AMR) is a significant global public health concern, often linked to the overuse or improper use of antibiotics, including inadequate dosages, prolonged use, and multiple treatment rounds. AMR challenges for healthcare systems, balancing the growing number of infectious disease cases with limited antibiotic options [8]. The WHO’s Global Antimicrobial Resistance and Use Surveillance System (GLASS) and the 2015 Global Action Plan (GAP) call for standardized surveillance, reporting, antibiotic stewardship, and infection prevention [8,9]. GLASS data show AMR impacts 500,000 people across 22 countries and causes numerous deaths annually, with projections suggesting up to 10 million annual deaths by 2050 if resistance grows unchecked [10,11].

In India, the National AMR Containment Program (2012-2017), coordinated by the National Centre for Disease Control (NCDC) and the Indian Council of Medical Research (ICMR)’s Antimicrobial Resistance Surveillance and Research Network (AMRSN) since 2013, promotes rational antibiotic use [12]. In India, Schedule H1 regulation by the Central Drugs Standard Control Organisation (CDSCO) in 2014 prohibits over-the-counter antibiotic sales, yet enforcement remains inconsistent, allowing easy access [13,14]. AMR drives up healthcare costs with resistant infections requiring expensive broad-spectrum drugs and longer hospital stays, exacerbated by limited new antibiotic research [13]. In India, SMA is pervasive due to weak enforcement of prescription regulations, cultural reliance on pharmacists, and economic barriers, with studies reporting 32-54% prevalence [2,13]. This contributes to AMR, with 58,000 annual deaths from resistant infections and 30-60% resistance in key pathogens [14].

The COVID-19 pandemic amplified SMA in India, as restricted healthcare access drove reliance on over-the-counter drugs, underscoring the need for targeted research, particularly in North India, where data remains limited [15]. South-East Delhi lacks targeted research, particularly post-COVID-19, when healthcare disruptions are likely to exacerbate SMA. This study aims to quantify SMA prevalence and evaluate the community's knowledge, attitudes, and practices in South-East Delhi, a region with unique urban-periurban dynamics. A qualitative component, employing focus group discussions, was included to explore the underlying reasons for SMA, such as trust in local chemists, economic constraints, and healthcare access barriers, which quantitative data alone cannot fully elucidate. This mixed-method approach provides a comprehensive understanding of SMA, informing tailored interventions to curb AMR in resource-constrained settings.

## Materials and methods

An explanatory sequential mixed-method study was conducted among adult residents of the field practice areas of a medical college (Pul Prahladpur and Madanpur Khader) in South-East Delhi from January 2023 to June 2024. This study included a quantitative component, utilizing a cross-sectional analytical study, which estimates the prevalence of SMA and its determinants, and a qualitative component, conducted through focus group discussions (FGDs) to explore the reasons for SMA among the study subjects. This design was chosen to combine statistical rigor with contextual depth, enabling comprehensive insights into SMA’s behavioral and structural determinants.

**Figure 1:**
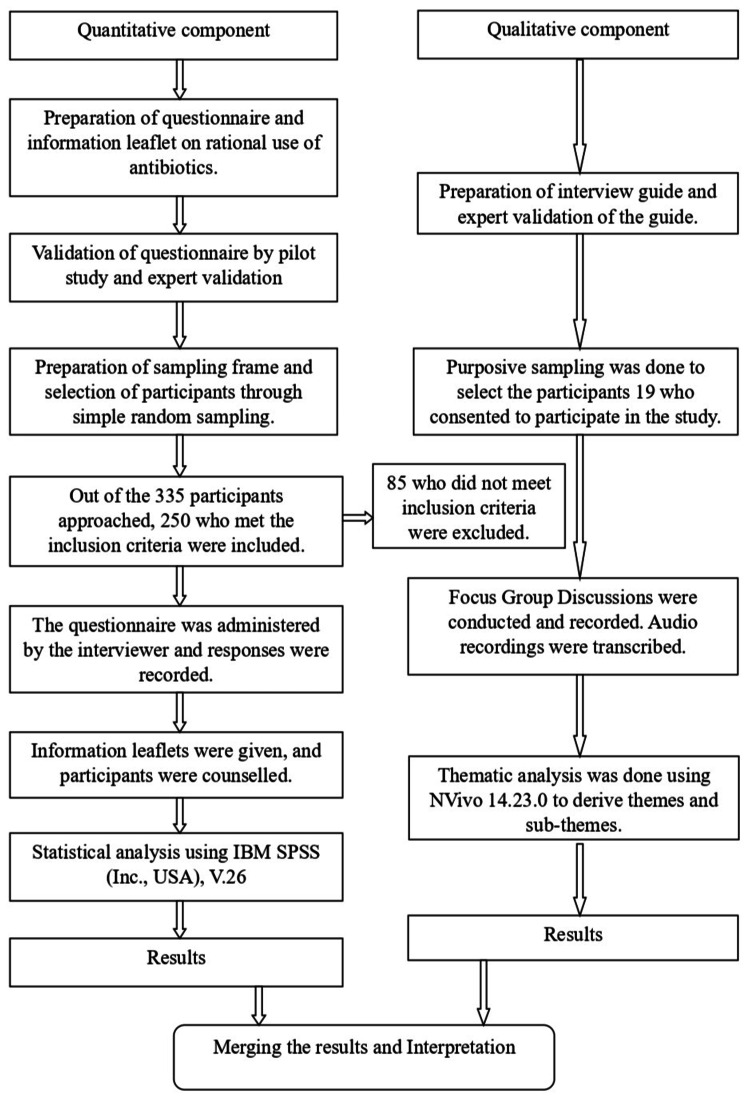
Schematic representation of methodology.

The study population comprised individuals aged ≥ 18 years, residents in the field practice areas of the urban and rural health training centers for ≥ 3 months, who self-reported antibiotic use in the past three months. Those with formal medical education were excluded from the study to avoid bias in knowledge, attitude, and practice (KAP) responses. For the quantitative component of this study, a cross-sectional survey was conducted among 250 adults, selected via simple random sampling, to assess SMA. The sample size was calculated based on the prevalence of SMA (17%) among the residents of urban slums located near Government Medical College, Jabalpur, Madhya Pradesh [16]. The sample size of 250 was calculated with an absolute error of 5% and a 95% confidence interval. Nineteen subjects for the FGDs were chosen via purposive sampling (Figure 1).

The study was approved by the Institutional Ethics Committee of Hamdard Institute of Medical Sciences and Research, before data collection. Written informed consent was obtained from all participants after explaining the study’s purpose, procedures, confidentiality, and voluntary nature, with the right to withdraw. All data were anonymized to protect participant privacy.

In the first phase, data collection was conducted using a self-designed, pre-tested, and validated (by two pharmacology experts and supervisors) structured questionnaire through face-to-face interviews during the daytime. This process took approximately 30 minutes per participant. The questionnaire contained 51 questions categorized into three sections: Section A solicited personal information and socio-demographic data, Section B comprised questions assessing the knowledge of participants about antibiotics, antibiotic resistance, and Section C gathered information on attitude, practice, and pattern of SMA over the past three months. To validate SMA, the participants were asked to provide the leftover antibiotic strips or packages, bills, old prescriptions used, or any electronic advice, such as WhatsApp or text messages. The questionnaire was piloted on 10% of the study participants before the actual data collection, and data from the pretest were excluded from the final analysis. Participants received AMR education pamphlets post-interview.

In the second phase, FGDs were conducted by the principal investigator (female), who was trained in qualitative research. After obtaining written informed consent, a self-designed guide with open-ended questions was used. The guide was pilot-tested before the start of the actual study. Three FGDs, each lasting about 60 minutes, were held with 19 participants in groups of six to seven each, at their respective urban health training center (UHTC) or rural health training center (RHTC). After the third FGD, data saturation was achieved as no new information emerged, and the point of redundancy was attained. Audio recordings ensured accuracy, with an observer creating sociograms to track group dynamics. Privacy and confidentiality were maintained.

The data was entered into MS Excel and analysed using SPSS (IBM Corp. Released 2019. IBM SPSS Statistics for Windows, Version 26.0. Armonk, NY: IBM Corp). Descriptive statistics were employed to describe the quantitative results. Categorical data were presented as frequencies and percentages. Modified Bloom’s cut point was used to calculate the score of knowledge, attitude, and practices of antibiotic use and SMA. The association between independent variables and SMA was tested using Pearson’s Chi-squared test and Fisher’s exact test. Binary logistic regression at 95% CI was computed to demonstrate the significant associations between the independent variables and SMA, with statistical significance set at a p-value <0.05.

For the qualitative component, data were analyzed using thematic analysis. Audio recordings of each FGD were transcribed by a transcriber who has proficiency in the Hindi language and then translated into English. It was further back-translated by the principal investigator (PI). Each transcript was double-checked for accuracy. These cross-checking and confirmation procedures were done to ensure that the quality of the work was maintained. Open coding was done by the PI to capture in vivo perspectives, and the data were grouped into categories with selective themes and sub-themes derived inductively through thematic analysis. NVivo 14 (Lumivero 2023, NVivo Version 14, Denver) was used for the same. Reflexivity was maintained by incorporating field notes, team debriefings, and member checking. To enhance the accuracy of the results, the data and themes were verified by research team members trained in qualitative analysis. The preliminary results were shared with participants to ensure that interpretations accurately reflected their perspectives. This verification process ensured the credibility, transferability, dependability, and conformability of the qualitative data [17].

## Results

Quantitative results

The mean age of the participants was 36.3±11.4 years, with 93 (37.2%) aged 18-30 years. A hundred and forty-eight (59.2%) of the sample were females, with 121 (48.4%) among them being non-pregnant and non-lactating, and the remaining 27 (10.8%) being pregnant or lactating. Most of the participants, i.e., 193 (77.2%), were married, 46 (18.45%) were never married, and the rest were widowed/divorced. A hundred and eighty-one (72.4%) had high school or higher, and 161 (64.4%) were employed. 117 (46.8%) belonged to the upper middle socioeconomic class (according to the Modified Kuppuswamy Scale, 2023) (Table 1).

**Table 1:** Sociodemographic characteristics of the study participants (N=250).

Variable	Frequency	Percentage
Age		
18-30 years	93	37.20%
31-40 years	83	33.20%
41-50 years	42	16.80%
51-60 years	29	11.60%
>60 years	3	1.20%
Gender		
Male	102	40.80%
Female	148	59.20%
Marital Status		
Married	193	77.20%
Never Married	46	18.45%
Widowed	9	3.60%
Divorced	2	0.80%
Religion		
Hinduism	143	57.20%
Islam	81	32.40%
Christianity	26	10.40%
Caste		
Other backward classes	102	53.20%
General	99	18.80%
Scheduled caste/ Scheduled tribe	31	20.80%
Don’t know	18	7.20%
Education level		
Primary School	37	14.80%
Middle School	32	12.80%
High School	51	20.40%
Intermediate/ Diploma	64	25.60%
College/ University	59	23.60%
Professional	7	2.80%
Employment Status		
Paid employment	161	64.40%
Unemployed*	89	35.60%
Socioeconomic class		
Upper	0	0.00%
Upper middle	117	46.80%
Lower middle	81	32.40%
Upper lower	52	20.80%
Lower	0	0.00%
Residence		
Field practice area of the Rural Health Training Centre	150	60.00%
Field practice area of the Urban Health Training Centre	100	40.00%
Chronic disease		
Yes	37	40.65%
No	54	59.35%

Among 250 participants, 91 (36.4%, {95% CI: 31.4-41.4}) practiced SMA in the past three months. Of these, 63 (69.2%) self-medicated once, 22 (24.2%) twice, and the rest more than twice. Among females who practiced SMA, 48 (39.3%) were non-pregnant/non-lactating women, while 7 (25.43%) were pregnant or lactating women. Common indications for SMA were cold and flu-like symptoms (29, 31.86%), fever (27, 29.69%), burning micturition/dysuria (15, 16.48%), sore throat (13, 14.28%), and pain (7, 7.69%). SMA was more common among individuals without chronic diseases (54, 59.35%) than those with chronic diseases (37, 40.65%), including hypertension and diabetes mellitus. The primary sources of antibiotics for SMA were pharmacies (70, 67.3%), leftover antibiotics (27, 26%), and friends/family (7, 6.7%). The leading reasons for SMA included easy antibiotic availability (53, 58.24%), cost-saving (20, 21.97%), time-saving (16, 17.58%), and distrust in doctors (2, 2.21%). Adverse drug reactions (ADRs) were reported by 4 (4.4%) subjects who practiced SMA.

Among 91 participants who did SMA, the highest prevalence was in the 51-60 age group (13, 44.8%). SMA rates were similar among males (37, 36.3%) and females (54, 36.5%). Chi-square analysis found a significant association between marital status and SMA, with higher prevalence among married participants (78, 40.4%) than unmarried ones (13, 22.8%) (p<0.001). SMA was more common in rural health training center (RHTC) areas (59, 39.3%) than in urban health training center (UHTC) areas (32, 32%). Educational status significantly influenced SMA; 38 (55.1%) of those with education below or equal to high school practiced SMA compared to 53 (29.3%) of those with higher education (p<0.001). Socioeconomic status was also a significant factor; 28 (53.8%) of the upper-lower class, 47 (40.2%) of the upper-middle class, and 16 (19.8%) of the lower-middle class engaged in SMA (p<0.001). Additionally, SMA was significantly associated with the presence of chronic disease, with 37 (58.7%) of those with chronic illnesses practicing SMA compared to 54 (28.9%) without chronic disease (p<0.001).

A binary logistic regression model identified significant predictors of SMA, including marital status, religion, education, socioeconomic status, and chronic disease presence (Table 2). In the final model, being married, having an education below high school, belonging to the upper-middle or lower-middle class, and the absence of chronic disease significantly increased SMA risk.

**Table 2:** Sociodemographic factors associated with self-medication with antibiotics using binary logistic regression (N=250). OR: odds ratio; OBC: other backward classes; SC/ST- Scheduled caste/ Scheduled tribe; UHTC: urban health training center; RHTC: rural health training centre.

Variables	n	Self-medication with antibiotics		p-value		OR (95% CI)
		Yes (%)	No (%)			
Age						
18-30 years	93	27 (29%)	66 (71%)	Ref		
31-40 years	83	34 (41%)	49 (59%)	p=0.53		0.797(0.348-1.827)
41-50 years	42	16(38.1%)	26(61.9%)	p=0.953		1.033(0.352-3.029)
51-60 years	29	13(44.8%)	16(55.2%)	p=0.656		1.340(0.370-4.848)
>60 years	3	1 (33.3%)	2 (66.7%)	p=0.720		1.783(0.075-42.319)
Gender						
Male	102	37(36.3%)	65(63.7%)	Ref		
Female	148	54(36.5%)	94(63.5%)	p=0.293		1.569 (0.678-3.629)
Marital status						
Single	57	13(22.8%)	44(77.2%)	Ref		
Married	193	78(40.4%)	115(59.6%)	p=0.006		4.015 (1.488-10.832)
Religion						
Christianity	26	4(46.2%)	12(53.8%)	Ref		
Islam	81	28(34.6%)	53(65.4%)	p=0.001		14.805 (3.181-68.92)
Hinduism	143	49(34.3%)	94(65.7%)	p=0.030		3.741 (1.132-12.359)
Caste						
General	99	29(29.3%)	70(70.7%)	Ref		
OBC	102	47(46.1%)	55(53.9%)	p=0.001		0.133 (0.41-0.428)
SC/ST	31	9 (29.0%)	22(71.0%)	p=0.621		1.326 (0.433-4.058)
Others	18	6 (33.3%)	12(66.7%)	p=0.122		3.058 (0.742-12.602)
Employment status						
Employed	161	58 (36.02%)	103(63.98%)	p=0.859		1.089 (0.424-2.797)
Unemployed	89	33 (37.08%)	56 (69.92%)	Ref		
Area of Residence						
Field practice area of UHTC	100	32(32.0%)	68(68.0%)	Ref		
Field practice area of RHTC	150	59(39.3%)	91(60.7%)	p=0.236		1.550 (0.751-3.197)
Educational level						
Below high school	69	38(55.1%)	31(44.9%	p=0.043	2.950 (1.965-3.938)	
High school and above	181	53(29.3%)	128(70.7%)	Ref		
Socio-economic class						
Upper middle	117	47(40.2%)	70(59.8%)	p<0.001		4.310 (1.696- 10.956)
Lower middle	81	16(19.8%)	65(80.2%)	p<0.001		10.397(3.645-29.655)
Upper Lower	52	28(53.8%)	24(46.2%)	Ref		
Presence of any chronic disease						
Yes	63	37(58.7%)	26(41.3%)	Ref		
No	187	54(28.9%)	133(71.1%)	p<0.001		5.289(2.211-12.652)

Age was not a significant predictor of SMA after adjusting for multiple variables. However, the odds of SMA increased with age [odds ratio OR) of (>60 years) = 1.783; 95% confidence interval (CI) of (0.075-42.319)]. Females had 1.57 times higher odds of SMA than males [OR = 1.569; 95% CI (0.678-3.629)]. Married participants were more likely to practice SMA than single individuals. Employed individuals were more likely to engage in SMA [OR = 1.089; 95% CI (0.424-2.797)]. Residents of RHTC areas had 1.5 times higher odds of SMA than UHTC residents. Participants with lower education had higher SMA odds. The upper-middle and lower-middle classes had 4.3- and 10.4 times higher odds of SMA than the upper-lower class. Participants without chronic diseases were more likely to practice SMA than those with chronic illnesses.

**Figure 2:**
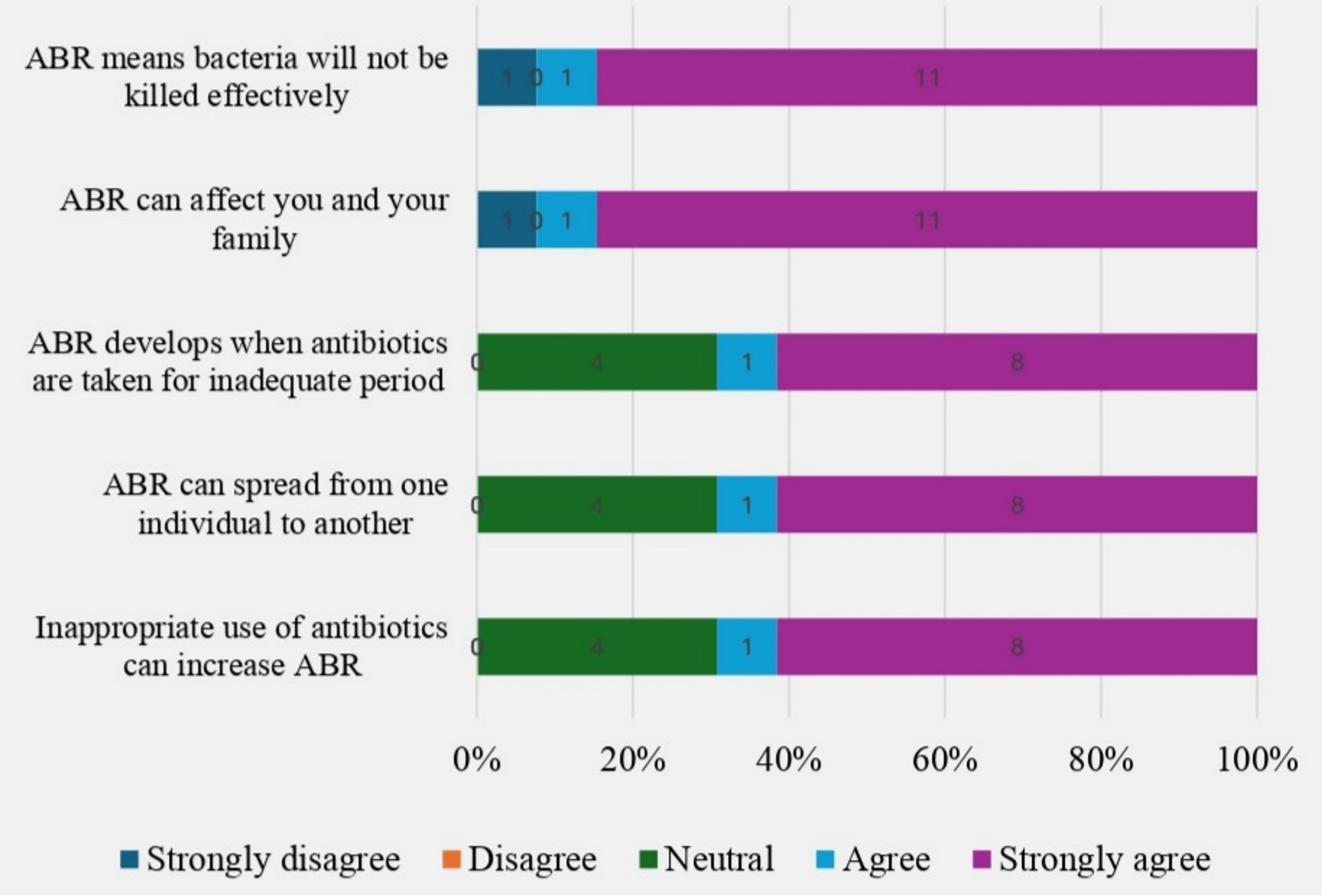
Knowledge of participants regarding antibiotic resistance (n=13). ABR: antibiotic resistance.

Knowledge was assessed using six questions (Table 3). Correct answers received a score of one, with a maximum of six points. Using the Modified Bloom's cut-off point, scores ≥5 (≥80%) indicated adequate knowledge, while scores <5 (<80%) were inadequate. While 218 (53.8%) correctly identified antibiotics as used for bacterial infections, only 178 (48.6%) understood their function. A significant portion (148, 59.2%) mistakenly believed antibiotics work against viral infections. However, 152 (60.8%) knew antibiotics are generally unnecessary for most coughs and colds, 188 (75.2%) for most diarrhea cases, and 191 (76.4%) correctly distinguished antibiotics from antipyretics like paracetamol. Overall, 167 (66.8%) had inadequate knowledge about antibiotics. SMA was significantly associated with poor knowledge (p<0.05). Only 13 (5.2%) of participants were aware of AMR, while 237 (94.8%) were unaware. Awareness of AMR was significantly associated with SMA (p<0.05) (Figure 2).

**Table 3:** Knowledge of study participants about antibiotics and their use (N=250). *multiple-choice question with a single best response.

Questions assessing knowledge about antibiotics and their use	Correct response (%)	Incorrect response (%)
What are antibiotics prescribed for?^ *^	218 (53.8%)	187 (46.2%)
How do antibiotics function within body? ^*^	178 (48.6%)	188 (51.4%)
Are antibiotics effective against viral infections?^ *^	102 (40.8%)	148 (59.2%)
Are antibiotics needed in most cases of cough and cold?	152 (60.8%)	98 (39.2%)
Are antibiotics needed in most cases of loose motions?	188 (75.2%)	62 (24.8%)
Are antibiotics similar to fever medicines and painkillers?	191 (76.4%)	59 (23.6%)

Regarding attitudes, 163 (65.2%) disagreed with obtaining antibiotics from friends/relatives without a prescription, and 144 (57.6%) disagreed with acquiring them from a pharmacy without a prescription. Additionally, 173 (69.2%) disagreed with keeping antibiotics at home for future use. Overall, 142 (56.8%) had a conducive attitude toward appropriate antibiotic use, while 108 (43.2%) had a non-conducive attitude. Attitude was significantly associated with SMA (p<0.05).

Three questions assessed SMA practices. Correct responses scored one point, with total scores ranging from 0 to three. Using the Modified Bloom's cut-off, scores of 3 (≥80%) were considered appropriate, while scores <3 (<80%) indicated inappropriate practices (Table 4). A hundred and eighty-five (74%) reported completing their last course, while 65 (26%) did not. About 222(88.8%) reported no dosage alteration, while 28 (11.2%) admitted to modifying it. About 241 (96.4%) adhered to prescriptions, while 9 (3.6%) changed antibiotics during treatment. Overall, 171 (68.4%) adhered to appropriate antibiotic use practices, while 79 (31.6%) exhibited inappropriate practices. No significant association was found between practice and SMA.

**Table 4:** Knowledge, attitude, and practice of study participants towards antibiotic use and self-medication with antibiotics classified using Modified Bloom's cutoff (N=250).

Knowledge	Frequency	Percentage (%)
Adequate (≥80%)	83	33.20%
Inadequate (<80%)	167	66.80%
Attitude		
Conducive (<80%)	142	56.8%
Non-conducive (≥ 80%)	108	43.2%
Practice		
Appropriate (≥ 80%)	171	68.4%
Inappropriate (<80%)	79	31.6%

Qualitative results

**Figure 3:**
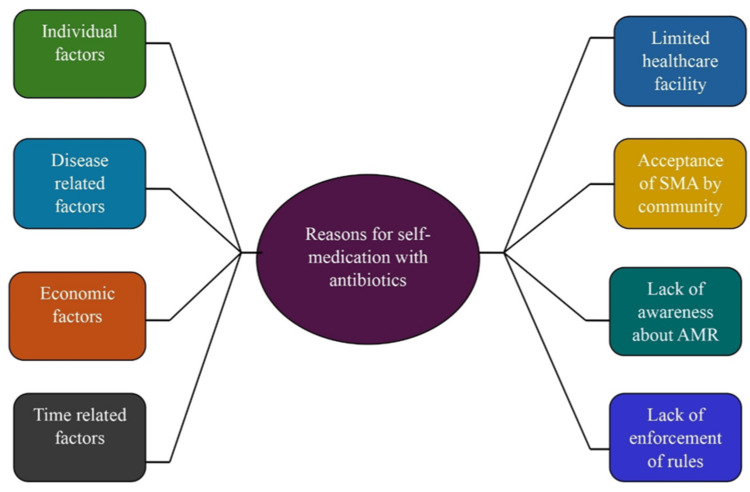
Major reasons that lead to self-medication with antibiotics by the participants.

Inductive thematic analysis revealed several themes (Figure 3, Table 5).

**Table 5:** Themes and subthemes derived from codes and selected participant quotes (N=19). FGD: focus group discussion.

Themes	Subthemes	Participants' Quotations
Individual factors that motivate self-medication	Trust in local chemists	"Today in the YouTube era, people google their symptoms and go to the pharmacy and get medicines. Even chemists don’t ask for a prescription. This is a big fault." (36-year-old female, FGD 1)
	Past positive experiences	"If the same symptoms recur, then I’ll buy same medicines using old prescription." (45-year-old female, FGD 3)
	Peer advice and misconceptions	"The patient thinks his condition is not that severe and also, he accepts suggestions from anyone." (40-year-old male, FGD 1)
Disease-related factors	Neglectful attitude towards minor illnesses	"He gets medicines from the chemist but doesn’t know how many days to take. If he gets better, he will stop taking." (40-year-old male, FGD 1)
	Frequent recurrence of illnesses	"If the same symptoms recur, then I’ll buy same medicines using old prescription." (45-year-old female, FGD 3)
Economic drivers	Avoiding out-of-pocket expenses	"For coming and going it takes a lot of time. Also our job loss, we are working for daily wage, so my salary also will be cut." (40-year-old male, FGD 2)
	High cost of private healthcare	"Private hospitals charge a lot. For registration they take 200rs. It is too expensive." (38-year-old male, FGD 2)
Time-related factors	Long waiting times at government hospitals	"Nowadays people want to save that time if they go to the chemist, including a waiting period that takes 15 minutes." (32-year-old male, FGD 1)
	Clinic hours conflicting with work schedules	"We have a Mohalla clinic nearby... but their timing is from 10 AM to 1 PM. We have to go to work." (38-year-old male, FGD 3)
Limited healthcare accessibility	Distance to government hospitals	"We will get free treatment if we go to AIIMS, but too far. A lot of traffic on the way. One day complete waste if we go for fever and all." (45-year-old female, FGD 3)
	Convenience of nearby chemists	"Chemist shops are nearby. We can walk and go. Takes only 30 minutes hardly." (48-year-old male, FGD 3)
Community acceptance of self-medication	Casual attitude towards obtaining medicines	"People don’t bother much about where they go. They go to wherever they get services easily." (32-year-old female, FGD 2)
Lack of awareness about antimicrobial resistance	Misuse due to lack of knowledge	"By the time they go to the doctor, no use. Even if the doctor gives antibiotics, they don’t take them correctly." (31-year-old female, FGD 1)
	Incomplete antibiotic courses	"With only one tablet fever stopped. So I gave her tablets only for 2 days." (36-year-old female, FGD 2)
Lack of enforcement of regulations	Over-the-counter sale of antibiotics	"Why are chemists giving the medicines? They are not supposed to give such tablets with side effects without a prescription." (32-year-old female, FGD 3)
	Lack of legal consequences	"Yes, but no use. They don’t have fear. There is no law. Who will punish them? Poor people suffer." (35-year-old male, FGD 3)

Individual Factors

Participants self-medicate due to trust in chemists, past positive experiences, and peer advice. Misinformation from media and peers, combined with the easy availability of antibiotics, reinforces this behaviour. The perception that antibiotics provide quick relief discourages seeking medical consultation.

Disease-Related Factors

Minor illnesses are often considered unworthy of medical attention, leading individuals to rely on informal advice or past prescriptions. The recurrence of symptoms further encourages self-prescription without consulting doctors.

Economic Drivers

**Figure 4:**
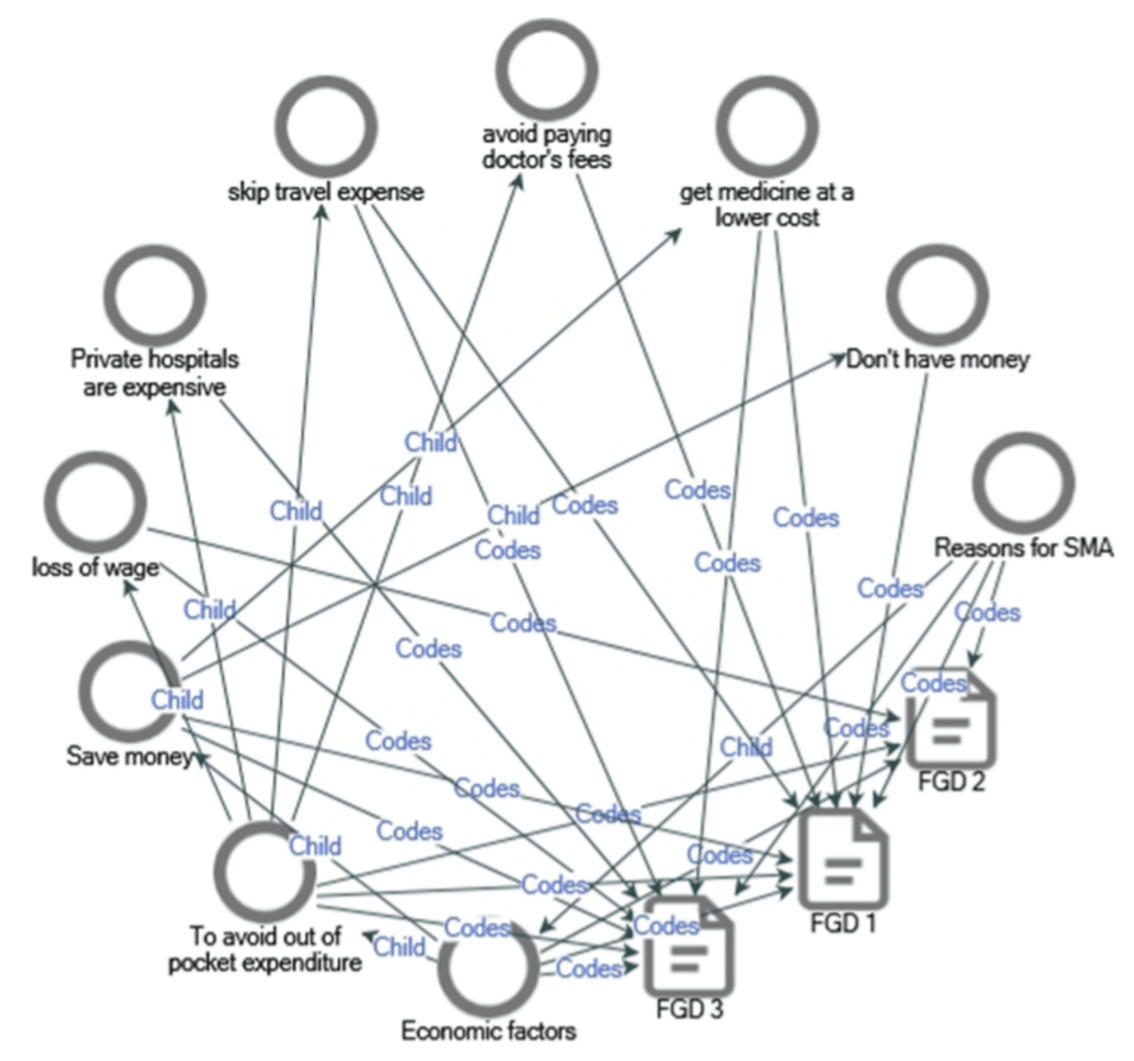
Project map depicting Economic factors that lead to self-medication with antibiotics (Source NVivo 14 plus output). Codes: represents a label or category, Child: indicates a subordinate code nested within a broader parent code.

Financial constraints significantly drive SMA. Participants avoid expenses such as consultation fees and travel costs. Daily wage workers fear income loss due to time spent in clinics. High private hospital fees further deter individuals from seeking formal healthcare (Figure 4).

Time Constraints

Long waiting times at government hospitals and restrictive clinic hours conflict with work schedules. The convenience of chemists, where medications can be obtained quickly without paperwork, makes them a preferred alternative.

Limited Healthcare Accessibility

The absence of nearby government hospitals and the limited operational hours of public healthcare facilities push individuals toward chemists. Despite the availability of free treatment at larger hospitals, the time and effort required discourage many from accessing them.

Community Acceptance

A casual attitude toward obtaining medicines reinforces SMA. Many prioritize ease and speed over legitimacy, believing chemists offer similar medications to doctors. This community-wide acceptance normalizes the practice of self-medication.

Lack of Awareness About AMR

A poor understanding of AMR leads to inappropriate antibiotic use. Many discontinue antibiotics once symptoms improve, unaware of the consequences. Informal providers and chemists contribute to this misuse by prescribing without proper medical supervision.

Weak Enforcement of Regulations

Despite regulations, chemists freely dispense antibiotics without prescriptions. The lack of strict enforcement and legal consequences further encourages self-medication, increasing the risk of antibiotic resistance.

Integration of findings

To enhance integration, a joint display (Table 6) was developed to synthesize the findings from both quantitative and qualitative strands. This integration reveals how the prevalence and patterns observed in the survey are supported and enriched by the in-depth narratives from focus group discussions. This integration provides a more nuanced understanding of the drivers of SMA in this context.

**Table 6:** Joint display of quantitative and qualitative findings on SMA drivers. SMA: self-medication with antibiotics; SES: socioeconomic status; AMR: antimicrobial resistance; FGD: focus group discussion; OTC: over-the-counter; IEC: information, education and communication.

Quantitative Finding	Qualitative Theme	Integration Insight	Implications for AMR Interventions
Marital status: Being married significantly predicts SMA (p<0.001).	Household responsibilities: FGDs revealed that married individuals self-medicate to quickly manage symptoms due to family obligations.	Married individuals’ time constraints drive SMA, aligning quantitative significance with qualitative convenience-seeking behavior.	Develop community workshops for married adults, emphasizing rational antibiotic use, delivered via local networks (e.g., Mahila Mandals)
Education: Lower education (<0.001).	Knowledge gaps: FGDs highlighted misconceptions (e.g., antibiotics for viral infections) among less-educated participants	Low education correlates with inadequate knowledge (66.8%), explaining higher SMA rates through qualitative reports of misuse	Launch educational campaigns in local languages, targeting less-educated communities, using visual aids to correct misconceptions.
SES: Higher SES (upper-middle/upper) predicts SMA (p<0.001).	Trust in chemists: FGDs showed wealthier individuals rely on pharmacists for quick access, assuming expertise.	Higher SES enables pharmacy access, with qualitative trust in chemists explaining quantitative SMA trends despite AMR unawareness (95%).	Implement pharmacist AMR training and Schedule H1 enforcement via audits to curb OTC sales, targeting urban pharmacies
Chronic disease: 41% of SMA users had chronic diseases (p<0.001).	Prior experience and reuse: FGDs noted that chronically ill participants reuse antibiotics based on prior prescriptions.	The statistical link is enriched by participants’ habitual reuse of known drugs for familiar symptoms, risking resistance and masking complications.	Promote telemedicine for chronic disease management, ensuring prescription adherence and reducing SMA reliance
Knowledge about antibiotics: 66.8% had inadequate knowledge, significantly associated with SMA (p<0.05).	Widespread misconceptions: Participants believed antibiotics cured viral infections; confused them with painkillers.	Quantitative knowledge gap is vividly reflected in participants’ beliefs and behaviors; lack of awareness contributes directly to SMA	Design targeted IEC campaigns on antibiotic use and AMR for community settings; include street plays, local influencers, and interactive media.
Knowledge about AMR: Only 5% of participants were aware of AMR (p<0.05).	Unawareness of consequences: Participants lacked understanding of AMR, importance of completing course, or future risks of misuse.	Severe knowledge deficit seen in both data strands; unawareness of AMR reflects in unsafe practices and complacency toward antibiotic misuse.	Integrate AMR education into school curricula and community health worker training; use real-life stories and consequences to drive urgency.

## Discussion

Discussion of the quantitative component

The demographic profile of our 250 participants (mean age: 36.3 ± 11.4 years, 72.2% with high school education or higher, 64.4% employed) shaped SMA patterns in South-East Delhi. More female participants were included, likely due to daytime interviews, aligning with similar studies [2,18]. Multivariate analysis identified being married (OR=2.1, 95% CI: 1.3-3.4) and lower education (OR=1.8, 95% CI: 1.1-2.9) as significant SMA predictors, consistent with a Kerala study (32% SMA prevalence, higher among less educated) [2]. Unlike a Northern India study reporting 54% SMA with higher unemployment [13,19], our employed majority suggests economic stability may not reduce SMA. These findings highlight married and less-educated adults as high-risk groups for targeted interventions.

The study areas were peri-urban, with 60% of participants from rural and 40% from urban health centers. The prevalence of SMA was 36.4% over the past three months, consistent with findings from studies in the UAE, but higher than those in China and Kerala [20-23]. Factors include high population density, cultural preferences, disparities in healthcare access, and informal healthcare providers. SMA rates (25.43%) among pregnant/ lactating women were lower than non-pregnant/non-lactating women (39.3%), possibly due to concerns about foetal health and regular antenatal visits.

Inadequate antibiotic knowledge was found in 66.8% of participants, influencing SMA behaviours. Nearly half were unaware that antibiotics treat bacterial infections, 51.4% did not understand their mechanism, and 59.2% mistakenly believed they work against viral infections. However, 60.8% correctly felt antibiotics were unnecessary for most coughs/colds, and 75.2% for diarrhoea cases. These findings align with other studies showing significant knowledge gaps about antibiotics [24,25].

Among participants with a positive attitude toward antibiotic use, SMA was more common. About 34.8% found it acceptable to obtain antibiotics without consultation, and 42.4% preferred pharmacies over prescriptions. Additionally, 30.8% stored antibiotics for future use. In contrast, a study linked negative attitudes to higher education levels [25].

Although 68% reported appropriate antibiotic use (74% completed courses, 88.8% did not alter doses), 38% still practiced SMA. This could stem from misreporting or using previous prescriptions. Many who preserved past prescriptions used them for SMA, and 9.6% used antibiotics prescribed for family members. Another study found that 60% of the participants had completed the antibiotic course, and 80% had adhered to doses, but 62% had stored leftover antibiotics [21].

Low awareness of AMR (5.2%), aligning with similar studies, highlights the need for public health interventions [26]. Awareness levels in different studies ranged from 38% in Romania to 54.7% in Saudi Arabia, likely due to sociodemographic differences and varying knowledge levels about antibiotic use [21,27].

Sociodemographic factors significantly associated with SMA included marital status, religion, education, socioeconomic status, and chronic diseases. Nearly half of SMA users were aged 51-60, though younger age groups had higher prevalence in some studies [26,28]. Older adults may self-medicate due to access to leftover antibiotics and chronic disease management.

About 41% of SMA users had chronic diseases (p<0.001), contradicting findings from Bangladesh, where SMA was much higher in chronically ill individuals who were familiar with treatments [29]. Multivariate analysis confirmed chronic disease as an SMA predictor.

Gender differences were noted, with women more likely to self-medicate, though findings were not statistically significant. Married individuals had higher SMA prevalence, possibly due to recurrent infections and cultural stigma, contrasting with Ethiopian studies showing higher SMA among widows. Vulnerable groups like widows, facing economic and access barriers, may exhibit similar SMA patterns, as seen in other low-resource settings [30]. Unemployed participants had slightly higher SMA rates, consistent with other studies, possibly due to financial constraints leading to cost-effective alternatives like SMA.

Residents of rural health centres practiced SMA more (39.3%) than urban residents, likely due to limited healthcare access and over-the-counter antibiotic availability. Lower education levels were linked to higher SMA prevalence, consistent with studies where lack of knowledge contributed to misuse. However, a Kerala study found higher education associated with SMA, likely due to confidence in self-diagnosis [28,31].

Socioeconomic status (SES) was significantly associated with SMA (p<0.001), with regression analysis showing higher SES classes (upper-middle/upper) had increased odds compared to the upper-lower class, which had the highest prevalence. This aligns with a Kerala study (32% SMA, higher among educated, wealthier groups) [2], reflecting greater access to pharmacies and confidence in self-diagnosis, as seen in our FGDs’ - trust in chemists. Conversely, a Northern India study (54% SMA) found that lower SES drove SMA for cost-saving [13]. Higher SES reliance on pharmacists, despite AMR unawareness (95%), underscores the need for AMR education [2].

Inadequate knowledge and a favourable attitude toward SMA were significantly associated with higher SMA prevalence. About 50% of those with inadequate knowledge engaged in SMA, compared to 22.9% with adequate knowledge. Knowledge gaps about AMR were striking, with no SMA users correctly answering AMR-related questions [25,32].

Participants with a conducive attitude towards SMA (43%) were more likely to engage in SMA than those with a non-conducive attitude (27.8%). Misconceptions about antibiotics and a lack of awareness contributed to SMA, though some studies reported negative attitudes correlating with SMA, likely due to educational differences [25]. Lack of awareness about antimicrobial resistance was significantly associated with SMA, highlighting the need for targeted public health interventions to address these gaps and reduce the prevalence of SMA.

Discussion of the qualitative component

Focus group discussions revealed multiple SMA drivers, including individual perceptions, misinformation, limited healthcare access, economic constraints, time pressures, and community norms.

Participants widely accepted SMA, often bypassing doctors due to a lack of awareness about antibiotic rational use and potential consequences. This aligns with global studies showing misinformation and poor awareness as key SMA drivers [33].

Trust in chemists over formal healthcare providers was a major factor, consistent with findings from low- and middle-income countries where pharmacists’ accessibility and familiarity drive SMA [34]. Distrust in doctors and reliance on past experiences and peer advice further exacerbated SMA [33].

A widespread lack of antibiotic knowledge was evident, with antibiotics often misused for viral illnesses or considered pain relievers. Easy access, compounded by misinformation from media and peers, reinforced this behaviour, similar to studies in the UAE, Mozambique, and Indonesia [35-37].

Limited public healthcare accessibility, long waiting times, and repeated visits discouraged formal care-seeking, aligning with rural and urban Indian studies [38]. Cost constraints further influenced SMA, as participants sought affordable alternatives, mirroring findings from global research [33-35]. Time efficiency was another significant factor, as participants favoured quick access to antibiotics from chemists, a common trend worldwide [33,34]. Limited operating hours of government clinics and the absence of nearby hospitals were significant barriers, reinforcing the preference for SMA. This issue was highlighted in studies from various low and middle-income countries, where the lack of accessible healthcare facilities leads to increased self-medication [34].

A concerning knowledge gap among pharmacy staff was noted, particularly regarding antibiotic side effects. Weak regulatory mechanisms and insufficient penalties encourage non-compliance, highlighting the need for stricter enforcement and public education [33,37].

In South-East Delhi, the lack of enforcement of regulations, such as the Drugs and Cosmetics Rules, 1945, and Schedule H1 (2013), facilitates the over-the-counter dispensing of antibiotics without prescriptions were significant issues identified. Our findings highlight pharmacists’ role, with FGDs revealing trust in chemists despite 66.8% inadequate antibiotic knowledge. Policy should strengthen Schedule H1 enforcement through regular pharmacy audits and penalties, as seen in Kerala’s reduced SMA (32%) post-regulation [2]. Practice changes include mandatory pharmacist training on AMR risks and public campaigns targeting high-risk groups. It is a shared responsibility of the government, pharmacists, and the public to ensure compliance with these regulations.

Overall, SMA stems from cultural norms, trust in chemists, economic and time constraints, and healthcare inaccessibility. Strengthening regulations, improving healthcare access, and enhancing public awareness are essential to combat SMA and prevent AMR proliferation.

Strengths and limitations

Strengths of the Study

This pioneering community-based mixed-method study in South-East Delhi provided critical baseline data on SMA, using binary logistic regression and FGDs with 19 participants to offer deep socio-cultural insights. The mixed-method design, pilot-tested instruments, and researcher training ensured methodological rigor, while educational pamphlet distribution laid the groundwork for future public health interventions.

Limitations of the Study

The cross-sectional design limited causal inference, and the focus on South-East Delhi’s urban-periurban areas restricted generalizability. Self-reported data over three months introduced recall bias, while purposive sampling in FGDs risked selection bias, potentially skewing representativeness. Social desirability bias, qualitative subjectivity, and dominant voices in FGDs may have affected data authenticity, and excluding pharmacists’ and healthcare providers’ perspectives limited a comprehensive understanding of SMA drivers.

## Conclusions

This mixed-method study in South-East Delhi’s medical college field areas reveals a 36.4% prevalence of SMA among 250 adults, driven by easy availability, cost-saving, and convenience, primarily via unregulated pharmacy purchases. SMA, targeting colds, fevers, and urinary symptoms, significantly correlates with marital status, lower education, upper-middle/lower-middle socioeconomic class, and absence of chronic diseases (p<0.05). Alarmingly, 66.8% lack adequate antibiotic knowledge, and 95% are unaware of AMR, heightening risks of resistant pathogens, morbidity, and reduced antibiotic efficacy. Qualitative insights highlight trust in chemists, pharmacy convenience, and limited healthcare access as key drivers, possibly worsened by COVID-19 disruptions.

Participants with favourable SMA attitudes were more likely to self-medicate, with knowledge and attitude strongly linked to the practice (p<0.05). This dual evidence underscores a public health crisis necessitating urgent action. Stricter enforcement of Schedule H1 to limit over-the-counter sales and improved healthcare access via extended clinic hours, telemedicine, and subsidized care is critical. Targeted community education must address high-risk groups- married, less-educated, and economically strained individuals- to correct misconceptions and promote rational antibiotic use. Community engagement through local leaders and technology integration (e.g., mobile apps) will promote rational antibiotic use. This study’s findings demand immediate, evidence-based interventions to curb SMA and AMR, offering a blueprint for policymakers globally. By tackling SMA’s structural and behavioural drivers, we can preserve antibiotic effectiveness, mitigate AMR’s escalating burden, and enhance health outcomes in India and similar settings, reinforcing the need for sustained, multifaceted strategies.

## References

[1] Bliss M (1999). William Osler: a life in medicine. https://archive.org/details/williamosler00mich.

[2] Rajendran A, Kulirankal KG, Rakesh PS, George S (2019). Prevalence and pattern of antibiotic self-medication practice in an urban population of Kerala, India: a cross-sectional study. Indian J Community Med.

[3] World Health Organization (2000). Guidelines for the regulatory assessment of medicinal products for use in self-medication. Geneva: World health Organization;2000. Available from. Guidelines for the regulatory assessment of medicinal products for use in self-medication.

[4] Limaye D, Limaye V, Fortwengel G, Krause G (2018). Self-medication practices in urban and rural areas of western India: a cross-sectional study. Int J Community Med Public Health.

[5] Núñez M, Tresierra-Ayala M, Gil-Olivares F (2016). Antibiotic self-medication in university students from Trujillo, Peru. Medicina Universitaria.

[6] Kotwani A, Joshi J, Lamkang AS (2021). Over-the-counter sale of antibiotics in india: a qualitative study of providers' perspectives across two states. Antibiotics (Basel).

[7] Juneja K, Chauhan A, Shree T (2024). Self-medication prevalence and associated factors among adult population in Northern India: a community-based cross-sectional study. SAGE Open Med.

[8] Klein EY, Van Boeckel TP, Martinez EM (2018). Global increase and geographic convergence in antibiotic consumption between 2000 and 2015. Proc Natl Acad Sci U S A.

[9] Patel IB, Balkrishnan R, Ahmad A (2012). Evaluation of self-medication antibiotics use pattern among patients attending community pharmacies in rural India, Uttar Pradesh. J Pharm Res.

[10] Laxminarayan R, Chaudhury RR (2016). Antibiotic resistance in India: drivers and opportunities for action. PLoS Med.

[11] Antimicrobial Resistance Division (AMR), National Action Plans and Monitoring and Evaluation (NPM) (2015). Global Action Plan on Antimicrobial Resistance.

[12] (2021). The State of the World’s Antibiotics 2021: a global analysis of antimicrobial resistance and its drivers. Washington, DC: Center for Disease Dynamics, Economics & Policy. https://resistancemap.cddep.org/..

[13] de Kraker ME, Stewardson AJ, Harbarth S (2016). Will 10 million people die a year due to antimicrobial resistance by 2050?. PLoS Med.

[14] Walia K, Ohri VC, Mathai D (2015). Antimicrobial stewardship programme (AMSP) practices in India. Indian J Med Res.

[15] Sunny T, Jacob R, K K, Varghese S (2019). Self-medication: is a serious challenge to control antibiotic resistance?. Natl J Physiol Pharm Pharmacol.

[16] Susheela F, Goruntla N, Bhupalam PK, Veerabhadrappa KV, Sahithi B, Ishrar SM (2018). Assessment of knowledge, attitude, and practice toward responsible self-medication among students of pharmacy colleges located in Anantapur district, Andhra Pradesh, India. J Educ Health Promot.

[17] Ray I, Bardhan M, Hasan MM (2022). Over the counter drugs and self-medication: a worldwide paranoia and a troublesome situation in India during the COVID-19 pandemic. Ann Med Surg (Lond).

[18] Jain S, Thakur A, Peepre K, Kaushal S, Kasar P (2018). Prevalence of self-medication practices among the residents of urban slums located near govt. medical college, Jabalpur. Int J Community Med Public Health.

[19] Wertheim HF, Chuc NT, Punpuing S (2017). Community-level antibiotic access and use (ABACUS) in low- and middle-income countries: finding targets for social interventions to improve appropriate antimicrobial use - an observational multi-centre study. Wellcome Open Res.

[20] Al-Tarawneh A, Ali T, Al-Taani GM (2024). Public patterns and determinants of antibiotic self-medication and antibiotic knowledge in Southern Jordan. Antibiotics (Basel).

[21] Simegn W, Moges G (2022). Antibiotics self-medication practice and associated factors among residents in Dessie City, Northeast Ethiopia: community-based cross-sectional study. Patient Prefer Adherence.

[22] Alkhalifah HM, Alkhalifah KM, Alharthi AF, Elzahrany YR, Aljuhani MA (2022). Knowledge, attitude and practices towards antibiotic use among patients attending Al Wazarat Health Center. J Family Med Prim Care.

[23] Abduelkarem AR, Othman AM, Abuelkhair ZM, Ghazal MM, Alzouobi SB, El Zowalaty ME (2019). Prevalence of self-medication with antibiotics among residents in United Arab Emirates. Infect Drug Resist.

[24] Yin X, Mu K, Yang H (2021). Prevalence of self-medication with antibiotics and its related factors among Chinese residents: a cross-sectional study. Antimicrob Resist Infect Control.

[25] Ateshim Y, Bereket B, Major F (2019). Prevalence of self-medication with antibiotics and associated factors in the community of Asmara, Eritrea: a descriptive cross sectional survey. BMC Public Health.

[26] Awad AI, Aboud EA (2015). Knowledge, attitude and practice towards antibiotic use among the public in Kuwait. PLoS One.

[27] Nair D, Gayathri PV, Gopinath G (2024). Prevalence of self-medicated use of antibiotics among the population in Ernakulam District in Kerala, India. ECA.

[28] Topor G, Grosu IA, Ghiciuc CM, Strat AL, Lupuşoru CE (2017). Awareness about antibiotic resistance in a self-medication user group from Eastern Romania: a pilot study. PeerJ.

[29] Mohanlal J, Eliza SR, Nair A, Anilkumar A (2022). Antibiotic self-medication-prevalence and trends among adults attending an urban health centre in South Kerala. Int J Community Med Public Health.

[30] Mannan A, Chakma K, Dewan G (2024). Prevalence and determinants of antibiotics self-medication among indigenous people of Bangladesh: a cross-sectional study. BMJ Open.

[31] Demissie F, Ereso K, Paulos G (2022). Self-medication practice with antibiotics and its associated factors among community of Bule-Hora Town, South West Ethiopia. Drug Healthc Patient Saf.

[32] Aslam A, Zin CS, Jamshed S, Rahman NS, Ahmed SI, Pallós P, Gajdács M (2022). Self-Medication with antibiotics: prevalence, practices and related factors among the Pakistani public. Antibiotics (Basel).

[33] Elmahi RH, Alrasheed NA, Al Sayegh AH, Almobark AA, Banu N, Ali MD (2023). Knowledge, attitude, and practice of using antibiotics among the community in Eastern Province, Saudi Arabia. J Pharm Bioallied Sci.

[34] Janatolmakan M, Abdi A, Andayeshgar B, Soroush A, Khatony A (2022). The reasons for self-medication from the perspective of iranian nursing students: a qualitative study. Nurs Res Pract.

[35] Do NT, Vu HT, Nguyen CT (2021). Community-based antibiotic access and use in six low-income and middle-income countries: a mixed-method approach. Lancet Glob Health.

[36] Al-Kubaisi KA, De Ste Croix M, Vinson D, Sharif SI, Abduelkarem AR (2018). Erratum to: What drives using antibiotic without prescriptions? A qualitative interview study of university students in United Arab Emirates. Pharm Pract (Granada).

[37] Torres NF, Solomon VP, Middleton LE (2019). Patterns of self-medication with antibiotics in Maputo City: a qualitative study. Antimicrob Resist Infect Control.

[38] Karuniawati H, Hassali MAA, Suryawati S, Ismail WI, Taufik T, Wiladatika A (2020). Public practices towards antibiotics: a qualitative study. Clin Epidemiol Glob Health.

